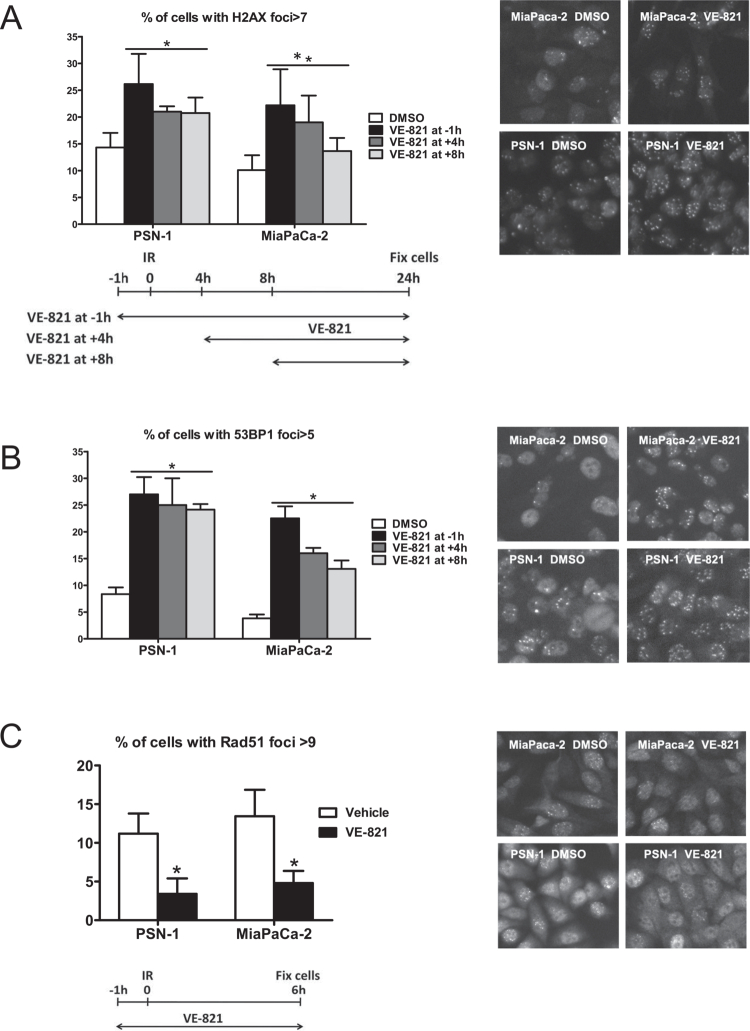# Correction

**DOI:** 10.1080/15384047.2025.2602376

**Published:** 2025-12-18

**Article title:** The novel ATR inhibitor VE-821 increases sensitivity of pancreatic cancer cells to radiation and chemotherapy

**Author:** Prevo, R., Fokas, E., Reaper, P. M., Charlton, P. A., Pollard, J. R., McKenna, W. G., … Brunner, T. B.

**Journal**: *Cancer Biology & Therapy*

**Bibliometrics**: Volume 13, Issue 11, pp. 1072–1081.

**DOI:** https://doi.org/10.4161/cbt.21093

In the original published article, the same image was inadvertently used in Figure 5C to represent the MiaPaca-2 VE-821 and PSN-1 VE-821 images. When concerns were raised regarding the similarities, the authors reviewed their data and noticed the error, working with the Publisher to rectify the issue. This error has no impact on the interpretation of the results, nor does it influence the conclusions of the work.

The authors apologize for any inconvenience that may have been caused.

**Figure 5.**